# Functional Oral Intake and Bowel Function in Enterally Fed Adults with Neurogenic Dysphagia: A Cross-Sectional Study

**DOI:** 10.3390/jcm15218529

**Published:** 2026-11-02

**Authors:** Burak Manay, Mustafa İbas, Hakan Parlak, Mehmet Nuri Elgörmüş, Mehmet Şerif Önen, Özcan Sönmez, Gizem İbas, Ramazan Ocal, Alperen Şentürk, Ramazan Güven

**Affiliations:** 1 Department of Speech and Language Therapy, Faculty of Health Sciences, Istanbul Atlas University, 34403 Istanbul, Turkey; mehmetnuri.elgormus@atlas.edu.tr (M.N.E.); alperen.senturk@atlas.edu.tr (A.Ş.); 2 Department of Otolaryngology—Head and Neck Surgery, Istanbul Atlas University, 34403 Istanbul, Turkey; mustafa.ibas@atlas.edu.tr; 3 Department of Anesthesiology and Reanimation, Faculty of Medicine, İstinye University, 34010 Istanbul, Turkey; hakan.parlak@istinye.edu.tr; 4 Department of Physical Medicine and Rehabilitation, Istanbul Atlas University, 34403 Istanbul, Turkey; serif.onen@atlas.edu.tr; 5 Department of Internal Medicine, Faculty of Medicine, Istanbul Atlas University, 34403 Istanbul, Turkey; ozcan.sonmez@atlas.edu.tr; 6 Department of Otolaryngology, Bağcılar Training and Research Hospital University of Health Sciences, 34200 Istanbul, Turkey; ibasgizem@gmail.com; 7 Department of Otolaryngology—Head and Neck Surgery, Ankara Training and Research Hospital, University of Health Sciences, 06230 Ankara, Turkey; drramazanocal@gmail.com; 8 Department of Emergency Medicine, Faculty of Medicine, Istanbul Atlas University, 34403 Istanbul, Turkey; ramazan.guven@atlas.edu.tr

**Keywords:** neurogenic dysphagia, enteral nutrition, Functional Oral Intake Scale, Bristol Stool Form Scale, bowel function

## Abstract

**Background/Objectives:** Gastrointestinal complications are common in enterally fed adults with neurogenic dysphagia. This study examined the association of functional oral intake with stool form and defecation frequency in this population. **Methods:** This prospective cross-sectional study included adults receiving enteral nutrition via a nasogastric tube or percutaneous endoscopic gastrostomy. Functional oral intake was assessed using the Functional Oral Intake Scale (FOIS), nutritional risk using the Nutritional Risk Screening 2002 (NRS-2002), and stool consistency using the Bristol Stool Form Scale (BSFS). Defecation frequency, daily fluid intake, enteral feeding duration, and feeding route were also recorded. Correlation and multivariable regression analyses were performed. **Results:** A total of 100 participants were included: 55 were classified as FOIS Level 1 and 45 as FOIS Levels 2–3. FOIS Level 1 was associated with hard versus normal stool (unadjusted odds ratio [OR] = 10.22, 95% confidence interval [CI]: 3.59–29.12; *p* < 0.001), with consistent adjusted and Firth estimates. The unadjusted association with soft/watery versus normal stool was nominally significant (OR = 3.17, 95% CI: 1.04–9.72; *p* = 0.043). Recorded fluid intake excluded formula water and was lower in FOIS Level 1, which entails no oral intake (*p* = 0.024). Defecation frequency differed between groups (*p* = 0.003). **Conclusions:** FOIS Level 1 was associated with hard stool, and defecation frequency differed between FOIS-defined feeding-status groups. Clinical differences between groups and unmeasured confounding limit interpretation. These findings are exploratory and hypothesis-generating and do not establish an independent or causal effect of FOIS status on bowel function.

## 1. Introduction

Dysphagia is a common complication of neurological disorders such as stroke, Parkinson’s disease, dementia, and traumatic brain injury, and has a substantial impact on nutritional status and clinical outcomes. By reducing or completely preventing oral intake, dysphagia increases the risk of malnutrition, dehydration, and aspiration pneumonia, and is associated with prolonged hospital stay, poorer functional recovery, and increased mortality [1,2,3]. Consequently, enteral nutrition has become a fundamental component of nutritional management in patients for whom oral feeding is unsafe or insufficient, ensuring adequate nutritional support while reducing the risk of malnutrition [4,5].

Although enteral nutrition is effective in maintaining nutritional status, it is frequently accompanied by gastrointestinal complications, including diarrhea, constipation, abdominal distension, and alterations in stool consistency [6,7,8]. The development of these complications is influenced not only by treatment-related factors, such as the route and duration of enteral feeding, enteral formula characteristics, and fluid intake but also by patient-related clinical characteristics [4]. Furthermore, accumulating evidence suggests that alterations in the enteric nervous system and the gut–brain axis in neurological disorders may impair gastrointestinal motility, indicating that dysphagia and bowel dysfunction may share common neurological and pathophysiological mechanisms [9].

Previous studies have primarily focused on enteral feeding methods, nutritional status, aspiration pneumonia, and swallowing function [4,5]. In contrast, the relationships between functional oral intake, nutritional risk, enteral feeding characteristics, and bowel function in patients with dysphagia receiving enteral nutrition have received considerably less attention. In particular, evidence comparing stool consistency and defecation patterns ‚between patients who are completely tube-fed and those with partial oral intake remains limited.

Therefore, the present study aimed to examine the association of functional oral intake with stool consistency and defecation frequency in enterally fed adults with neurogenic oropharyngeal dysphagia. Exploratory comparisons of clinical characteristics according to enteral feeding route (nasogastric tube versus percutaneous endoscopic gastrostomy) were also performed. We hypothesized that participants classified as Functional Oral Intake Scale (FOIS) Level 1 would have a higher prevalence of hard stool and less frequent defecation than those classified as FOIS Levels 2–3. The FOIS and feeding-route groups represented distinct functional feeding patterns and clinical populations; therefore, comparisons were intended to describe associations rather than establish causal or independent effects.

## 2. Methods

### 2.1. Study Design and Setting

This prospective cross-sectional observational study examined the association of functional oral intake with stool consistency and defecation frequency in enterally fed adults with neurogenic oropharyngeal dysphagia. The study protocol was approved by the Non-Interventional Scientific Research Ethics Committee of Istanbul Atlas University (Decision No. 26, Meeting No. 05, 11 May 2026), and written informed consent was obtained from all participants prior to enrolment. Following ethics committee approval, data collection was conducted between May and July 2026 at the Neurology and Physical Medicine and Rehabilitation clinics of Istanbul Atlas University Hospital.

Adult patients with clinically confirmed oropharyngeal dysphagia secondary to neurological disorders who were receiving enteral nutrition via a nasogastric (NG) tube or percutaneous endoscopic gastrostomy (PEG) were prospectively recruited. Data were collected through standardized clinical assessments, structured participant interviews, and review of medical records. Demographic characteristics, neurological diagnosis, functional oral intake level, nutritional risk, enteral feeding route and duration, stool consistency, defecation frequency, and daily fluid intake were recorded using standardized assessment forms. The STROBE checklist is provided in Supplementary File S1.

### 2.2. Study Population

The study population consisted of adult inpatients with oropharyngeal dysphagia secondary to neurological disorders receiving care in the Neurology and Physical Medicine and Rehabilitation services of Istanbul Atlas University Hospital. Neurological diagnoses included ischemic or hemorrhagic stroke, traumatic brain injury (TBI), Parkinson’s disease, dementia, and multiple sclerosis (MS).

The presence of an NG tube or PEG alone was not considered sufficient to establish dysphagia. Eligibility required a documented diagnosis established during routine clinical care by an otolaryngologist and a speech and language therapist and confirmed using a videofluoroscopic swallowing study (VFSS) or fiberoptic endoscopic evaluation of swallowing (FEES). Existing clinical and instrumental assessment reports were reviewed to confirm eligibility; no additional instrumental swallowing assessment was performed for this non-interventional study. All dysphagia diagnoses predated enrolment, although their timing relative to the initiation of enteral feeding could not be consistently established from the available records.

Eligible inpatients were prospectively recruited using consecutive sampling. All patients who met the eligibility criteria and provided written informed consent were enrolled sequentially during the study period. Inclusion criteria were age ≥ 18 years, documented neurogenic oropharyngeal dysphagia requiring enteral nutrition via an NG tube or PEG for at least one month, clinical stability, and a Mini-Mental State Examination (MMSE) score ≥ 24. All included participants met this cognitive eligibility criterion.

Participants with dementia had a pre-existing diagnosis documented in their hospital records and also met the MMSE ≥ 24 criterion. Dementia subtype and stage were not recorded and therefore could not be characterized. The MMSE threshold was used to facilitate participation in the assessments and reporting of bowel-related information; it was not used to exclude an existing dementia diagnosis or determine dementia stage. This criterion limits the applicability of the findings to patients with greater cognitive impairment.

Patients were excluded if they had dysphagia secondary to structural head and neck disease, including malignancy or previous surgery. Other exclusion criteria were gastrointestinal disorders known to directly affect bowel function, including inflammatory bowel disease, colorectal malignancy, intestinal obstruction, chronic diarrheal disorders, or previous major colorectal surgery; exclusive parenteral nutrition; active gastrointestinal infection or bleeding; abdominal surgery within the preceding three months; pregnancy; severe systemic illness requiring intensive care; incomplete clinical data; or inability to complete the assessments. Patients who had initiated or discontinued laxatives, antibiotics, probiotics, or prebiotics during the two weeks preceding enrolment were also excluded. Ongoing use of these treatments was not an exclusion criterion, and detailed chronic treatment information was unavailable for adjustment.

No formal a priori sample-size calculation was performed. During revision, a sensitivity power analysis was conducted for the association between the two FOIS groups and three stool-form categories using a noncentral chi-square approximation. The calculation corresponds to the G*Power version 3.1.9.7 settings “χ^2^ tests—Goodness-of-fit tests: Contingency tables,” with sensitivity analysis, N = 100, two degrees of freedom, α = 0.05, and 80% power. The detectable effect size was approximately Cohen’s w = 0.31. This calculation provides an approximate sensitivity benchmark for the unadjusted comparison but does not establish adequate power for the exact test or the adjusted multinomial and Firth logistic regression models. These regression analyses were therefore interpreted as exploratory, with emphasis on effect estimates and 95% confidence intervals.

### 2.3. Data Sources

Research data were collected prospectively through face-to-face clinical assessments, structured participant interviews, and review of electronic medical records.

Demographic and clinical characteristics, including age, sex, neurological diagnosis, enteral feeding route and duration, defecation frequency, Bristol Stool Form Scale (BSFS) score, and daily fluid intake, were recorded using a standardized data collection form developed by the researchers. Neurological diagnoses and relevant clinical information were confirmed using electronic medical records.

Information on stool form, defecation frequency, and daily fluid intake was obtained directly from participants through structured interviews and cross-checked against available nursing records and enteral feeding charts. However, the number of participants for whom each measure was cross-checked was not separately recorded; therefore, the proportion verified could not be reported.

### 2.4. Assessment Procedures

All assessments were performed by the same researcher using non-invasive methods under standardized clinical conditions during a clinically stable period while participants were receiving routine enteral nutrition. Face-to-face interviews were conducted to obtain information on usual bowel habits and daily fluid intake. Demographic and clinical information was obtained from participant interviews and electronic medical records and entered into standardized data collection forms.

Defecation frequency, enteral feeding route and duration, and reported daily fluid intake were also recorded. Recorded fluid volumes excluded water contained in enteral formulas.

All assessments were completed during a single evaluation session whenever possible.

#### 2.4.1. Functional Oral Intake Assessment (FOIS)

Functional oral intake level was assessed using the FOIS, a clinician-rated seven-level scale developed to evaluate oral feeding status in individuals with dysphagia [10]. FOIS Level 1 represents complete dependence on tube feeding with no oral intake. Level 2 represents tube dependence with minimal or inconsistent attempts at oral intake, whereas Level 3 represents tube dependence with consistent oral intake of food or liquid. FOIS ratings were assigned by the same researcher on the basis of each participant’s current feeding pattern, clinical evaluation, participant interview, and review of medical and feeding records. No additional standardized oral feeding trial or instrumental swallowing assessment was performed solely for FOIS scoring as part of the research protocol.

For the analyses, participants were categorized as FOIS Level 1 or FOIS Levels 2–3. Thus, the FOIS Levels 2–3 group comprised participants who continued to require enteral nutritional support but had some degree of oral intake, ranging from minimal swallowing trials to consistent oral intake supplemented by tube feeding. This grouping represented functional feeding status at the time of assessment and was not intended to serve as a direct measure of physiological dysphagia severity. The FOIS groups were regarded as clinically distinct functional feeding-status groups rather than directly comparable exposure groups. Comparisons between these groups were intended to describe observed patterns and not to estimate the independent or causal effect of FOIS status. The amount, frequency, consistency, and nutritional contribution of oral intake were not quantified separately within the FOIS Levels 2–3 group.

#### 2.4.2. Nutritional Risk Assessment (NRS-2002)

Nutritional risk was assessed using the Nutritional Risk Screening 2002 (NRS-2002), a screening tool developed by Kondrup and colleagues and recommended by the European Society for Clinical Nutrition and Metabolism (ESPEN) for identifying patients at risk of malnutrition [11].

The NRS-2002 evaluates nutritional risk based on nutritional status, including body mass index, recent weight loss, and reduced dietary intake, together with disease severity. In addition, 1 point is added for individuals aged 70 years or older. Total scores range from 0 to 7, and a score of ≥3 is considered indicative of nutritional risk [11].

The Turkish version of the NRS-2002 has demonstrated satisfactory validity and reliability in hospitalized medical and surgical patients [12]. In the present study, this version was administered according to the original scoring criteria. The dietary intake component was assessed using total nutritional intake, including both oral and enteral intake, rather than oral intake alone. Accordingly, the absence of oral intake in participants classified as FOIS Level 1 was not, by itself, considered evidence of reduced total nutritional intake.

#### 2.4.3. Stool Quality Assessment (Bristol Stool Form Scale)

Stool quality was assessed using the BSFS, a practical seven-category visual classification system developed by Lewis and Heaton to classify stool consistency [13]. The BSFS categorizes stool form from Type 1 (separate hard lumps) to Type 7 (watery stool). Because stool form is associated with colonic transit time, the BSFS is widely accepted as a simple and reliable clinical indicator of bowel function [13].

According to the scale, Types 1 and 2 represent hard stool forms commonly associated with constipation, Types 3 and 4 represent normal stool consistency, and Types 5–7 represent progressively softer or watery stools [13]. Although the BSFS is not intended to establish a diagnosis, it is widely used in both clinical practice and research to characterize bowel habits and stool consistency.

In the present study, participants identified the stool type that best represented their usual bowel pattern at the time of assessment. A fixed recall period was not specified. The BSFS was analyzed together with defecation frequency as an indicator of bowel function.

#### 2.4.4. Defecation Frequency Assessment

Defecation frequency was obtained through participant interviews and cross-checked against available nursing records. Participants were categorized according to their usual bowel frequency as once daily, every 2–3 days, once or twice weekly, or less than once weekly. A fixed recall period was not specified. This variable was evaluated alongside stool consistency to characterize bowel function.

#### 2.4.5. Daily Fluid Intake Assessment

Usual daily fluid intake was recorded in millilitres per day (mL/day) based on participants’ estimates of their average daily oral fluid intake and water administered through the feeding tube, including routine flushes. Reports were cross-checked against available nursing records and enteral feeding charts. For participants classified as FOIS Level 1, who had no oral intake, recorded volumes comprised only water administered through the feeding tube, including routine flushes. Water contained in enteral formulas was not quantified or included in the recorded volumes. Accordingly, this variable did not represent total daily water intake or an objective measure of hydration status and was partly linked to the FOIS-defined feeding status. Individual fluid requirements were not systematically assessed as part of the study; therefore, the adequacy of recorded intake relative to individual requirements could not be determined.

#### 2.4.6. Clinical and Enteral Feeding Characteristics

Enteral feeding characteristics included the route and duration of enteral nutrition. The feeding route was classified as NG tube or PEG according to the participant’s current nutritional support. Enteral feeding duration was calculated as the time elapsed from initiation of enteral nutrition to the study assessment and was recorded in months.

Neurological diagnoses, including stroke, TBI, Parkinson’s disease, dementia, and MS, were obtained from participants’ electronic medical records and confirmed during clinical evaluation.

### 2.5. Statistical Analysis

Descriptive analyses, group comparisons, correlation analysis, and multinomial logistic regression were performed using IBM SPSS Statistics (Version 30.0; IBM Corp., Armonk, NY, USA). Continuous and ordinal variables were summarized as mean ± standard deviation, minimum–maximum, and/or median (first–third quartiles), as appropriate. Categorical variables were presented as frequencies and percentages. Comparisons between FOIS groups and enteral feeding-route groups were performed using the Mann–Whitney U test. Daily fluid intake was compared across stool-form categories using the Kruskal–Wallis test. Categorical comparisons used Pearson’s chi-square or Fisher–Freeman–Halton exact tests, as appropriate. The association between daily fluid intake and BSFS score was assessed using Spearman’s rank correlation.

Stool form was classified as hard (BSFS Types 1–2), normal (Types 3–4), or soft/watery (Types 5–7). Because higher BSFS scores indicate progressively softer stool rather than consistently better bowel function, stool form was analyzed as a three-category outcome using multinomial logistic regression. Normal stool and FOIS Levels 2–3 were the reference categories.

Sequential models examined the association between FOIS group and stool form. Model 1 included FOIS group only; Model 2 additionally included age; and Model 3 additionally included daily fluid intake, expressed per 100 mL/day. The models contained two, four, and six slope coefficients, respectively, excluding intercepts. All three models included the same 100 participants, allowing comparison of FOIS estimates before and after adjustment for fluid intake. The adjusted models were simplified following concerns regarding sparse diagnostic categories and unstable estimates in the initial analyses.

The final multinomial models were additionally refitted in Python (version 3.12.14; Python Software Foundation, Beaverton, OR, USA) using statsmodels (version 0.15.0) for numerical verification. Convergence was assessed using the convergence status, score vector, and observed information matrix. Complete and quasi-complete separation were evaluated using linear programming implemented with SciPy (version 1.18.1). The refitted estimates were compared with the reported multinomial regression results.

Firth-penalized logistic regression was performed using the logistf package (version 1.26.0) in R (version 4.4.1; R Foundation for Statistical Computing, Vienna, Austria). as a sensitivity analysis to reduce small-sample bias and assess the consistency of the association under penalized estimation. Hard versus normal stool was examined in unadjusted and age-adjusted models, excluding participants with soft/watery stool (n = 81). An additional age-adjusted model compared hard stool with all other stool forms in the full sample (n = 100). Age was expressed per 10-year increase. Clinical diagnosis was omitted because of sparse diagnostic categories. Odds ratios (ORs) were reported with profile penalized-likelihood 95% confidence intervals (CIs), and *p* values were calculated using penalized likelihood-ratio tests. These analyses were considered exploratory and did not address unmeasured confounding.

An ordinal logistic regression model of defecation frequency was initially evaluated. However, the proportional-odds assumption was violated, and sparse cells limited estimation; therefore, this model was not retained for inferential interpretation. Defecation frequency was summarized descriptively and compared between FOIS groups using the Fisher–Freeman–Halton exact test.

Multicollinearity was assessed using tolerance and variance inflation factors (VIFs) calculated for the final model predictors: FOIS group, age, and daily fluid intake. Each predictor was regressed on the remaining predictors using an auxiliary linear regression with an intercept. These regressions were used solely for diagnostic purposes. VIF < 5 and tolerance > 0.20 were used as reference thresholds.

No missing observations were identified in the analyzed variables. Regression results were reported as odds ratios with 95% confidence intervals. Adjusted and secondary analyses were considered exploratory. No adjustment for multiple comparisons was applied, and secondary *p* values were interpreted cautiously. All tests were two-sided, with *p* < 0.05 used as the nominal significance threshold. The findings were interpreted as associations rather than causal effects.

## 3. Results

### 3.1. Participant Characteristics

A total of 100 enterally fed adults with oropharyngeal dysphagia were included in the study. Complete counts of patients screened, excluded, or declining participation were unavailable; therefore, a detailed screening flow could not be reconstructed. The mean age was 53.93 ± 17.30 years, and 66.0% of the participants were male. Stroke was the most common neurological diagnosis (36.0%), followed by traumatic brain injury (22.0%), dementia (20.0%), Parkinson’s disease (16.0%), and multiple sclerosis (6.0%).

Fifty-five participants (55.0%) were classified as FOIS Level 1, 24 (24.0%) as Level 2, and 21 (21.0%) as Level 3. For comparative analyses, Levels 2 and 3 were combined into a single group (n = 45; 45.0%). Enteral nutrition was provided through a PEG tube in 59.0% of participants and an NG tube in 41.0%. Daily defecation was reported by 43.0% of participants, whereas 24.0% defecated every 2–3 days, 20.0% once or twice weekly, and 13.0% less than once weekly.

According to the three-category stool-form classification, 38.0% of participants had hard stool (BSFS Types 1–2), 43.0% had normal stool (BSFS Types 3–4), and 19.0% had soft or watery stool (BSFS Types 5–7). The mean BSFS score was 3.14 ± 1.64, the mean NRS-2002 score was 3.85 ± 1.25, and the mean duration of enteral feeding was 3.59 ± 1.17 months. Nutritional risk, defined as an NRS-2002 score ≥ 3, was identified in 81 participants (81.0%). Mean daily fluid intake was 1365.00 ± 375.68 mL/day. Additional demographic and clinical characteristics are presented in Table 1.

### 3.2. Comparison of Continuous and Ordinal Variables According to FOIS Groups

No statistically significant differences in age or NRS-2002 score were observed between the FOIS groups (both *p* > 0.05). Participants classified as FOIS Level 1 had a longer duration of enteral feeding than those classified as FOIS Levels 2–3 (median [Q1–Q3]: 4 [3–5] vs. 3 [2–4] months; *p* < 0.001). They also had lower BSFS scores (2 [1–4] vs. 4 [3, 4]; *p* = 0.002), indicating a shift toward harder stool forms. Daily fluid intake was lower in the FOIS Level 1 group (median: 1250 vs. 1400 mL/day; *p* = 0.024). Between-group comparisons were performed using the Mann–Whitney U test. Detailed results are presented in Table 2.

### 3.3. Comparison of Categorical Variables According to FOIS Groups

Categorical variables were compared between the FOIS groups using Pearson’s chi-square test or the Fisher–Freeman–Halton exact test, as appropriate. Statistically significant associations were observed between FOIS group and enteral feeding route (χ^2^ = 5.145, *p* = 0.023), defecation frequency (exact *p* = 0.003), and stool-form classification (exact *p* < 0.001).

Participants classified as FOIS Level 1 more frequently received PEG feeding than those classified as FOIS Levels 2–3 (69.1% vs. 46.7%). Daily defecation was more frequent in the FOIS Levels 2–3 group than in the FOIS Level 1 group (60.0% vs. 29.1%).

Hard stool (BSFS Types 1–2) was reported in 56.4% of participants classified as FOIS Level 1 and 15.6% of those classified as FOIS Levels 2–3. Normal stool (BSFS Types 3–4) was reported in 23.6% and 66.7%, respectively, whereas soft or watery stool (BSFS Types 5–7) was reported in 20.0% and 17.8%, respectively.

No statistically significant associations were observed between FOIS group and sex (*p* = 0.581) or clinical diagnosis (exact *p* = 0.132). Detailed results are presented in Table 3.

### 3.4. Daily Fluid Intake and Stool Form

Recorded daily fluid intake was lower in participants classified as FOIS Level 1 than in those classified as FOIS Levels 2–3 (1280.0 ± 326.1 vs. 1468.9 ± 408.6 mL/day; Mann–Whitney U test, *p* = 0.024). Recorded volumes included oral fluids and water administered through the feeding tube but excluded water contained in enteral formulas. Because participants at FOIS Level 1 had no oral intake, this comparison partly reflected the feeding-group definitions and did not represent a comparison of total water intake. Mean recorded daily fluid intake was 1253.9 ± 323.9 mL/day in the hard-stool group, 1450.0 ± 432.0 mL/day in the normal-stool group, and 1394.7 ± 284.3 mL/day in the soft/watery-stool group. The overall difference between stool-form categories was not statistically significant (Kruskal–Wallis test, *p* = 0.098). No statistically significant correlation was observed between recorded daily fluid intake and BSFS score (Spearman’s ρ = 0.131, *p* = 0.194). These exploratory results are presented in Table 4.

### 3.5. Comparison of Clinical Characteristics According to Enteral Feeding Route

Participants receiving enteral nutrition via PEG were older than those receiving nutrition through an NG tube (median [Q1–Q3]: 61 [45.5–69.5] vs. 53 [32–63] years; *p* = 0.034). No statistically significant differences were observed between the groups in NRS-2002 score, duration of enteral feeding, BSFS score, or daily fluid intake (all *p* > 0.05). Between-group comparisons were performed using the Mann–Whitney U test. Detailed results are presented in Table 5.

### 3.6. Multicollinearity Diagnostics

Multicollinearity was assessed using tolerance and variance inflation factors calculated for the final model predictors: FOIS group, age, and daily fluid intake. Auxiliary linear regressions were used solely for diagnostic purposes; their coefficients were not interpreted as associations with bowel outcomes.

VIF values ranged from 1.048 to 1.119, and tolerance values ranged from 0.894 to 0.954, indicating no evidence of problematic multicollinearity among the final model predictors. Detailed results are presented in Table 6.

### 3.7. Association Between FOIS Group and Stool-Form Categories

In the unadjusted model, FOIS Level 1 was associated with higher odds of hard versus normal stool compared with FOIS Levels 2–3 (OR = 10.22, 95% CI: 3.59–29.12; *p* < 0.001). The estimate changed little after adjustment for age (OR = 10.33, 95% CI: 3.61–29.55; *p* < 0.001). Following additional adjustment for daily fluid intake, the OR decreased to 8.93 (95% CI: 3.07–25.97; *p* < 0.001). Daily fluid intake was not significantly associated with hard versus normal stool (OR = 0.887 per 100 mL/day, 95% CI: 0.763–1.032; *p* = 0.121).

For soft/watery versus normal stool, the association with FOIS group was statistically significant in Models 1 and 2 but did not reach statistical significance after adjustment for fluid intake in Model 3 (OR = 3.13, 95% CI: 0.98–9.98; *p* = 0.053). These adjusted analyses were exploratory and do not establish causal effects or eliminate unmeasured confounding. Detailed results are presented in Table 7.

All three final multinomial models converged, with finite coefficient estimates and standard errors and positive-definite observed information matrices. No complete or quasi-complete separation was detected. Additional refitting reproduced the reported estimates within rounding precision.

### 3.8. Sensitivity Analyses

In the unadjusted Firth-penalized logistic regression analysis, FOIS Level 1 was associated with higher odds of hard versus normal stool (OR = 9.49, 95% CI: 3.56–27.95; *p* < 0.001). After adjustment for age, the association remained similar (OR = 9.38, 95% CI: 3.51–27.93; *p* < 0.001). When hard stool was compared with all other stool forms in the full sample, the age-adjusted OR was 6.46 (95% CI: 2.64–17.51; *p* < 0.001). These sensitivity analyses supported the direction of the primary association but did not address unmeasured confounding. Detailed results are presented in Table 8.

## 4. Discussion

The present study examined functional oral intake and bowel function in enterally fed adults with neurogenic dysphagia. Participants classified as FOIS Level 1 more frequently reported hard stool than those classified as FOIS Levels 2–3. The association with hard versus normal stool persisted after adjustment for age and daily fluid intake, with similar findings in Firth sensitivity analyses. Adjustment for fluid intake attenuated the association, although its contribution remains uncertain. For soft or watery versus normal stool, the association was statistically significant in the unadjusted and age-adjusted models but not after additional adjustment for fluid intake. Daily fluid intake was lower in the FOIS Level 1 group, and defecation frequency differed between FOIS groups. These findings should be interpreted as exploratory given the limited precision of estimates, clinical differences between groups, and unmeasured confounding.

The observed between-group differences in stool consistency may reflect the combined influence of physiological, nutritional, and clinical factors. Reduced oral intake, inadequate fluid and fibre consumption, prolonged dependence on enteral nutrition, and decreased mobility may contribute to delayed intestinal transit and harder stool consistency in patients with dysphagia [14]. Our findings are consistent with previous studies reporting that gastrointestinal complications are common among individuals with neurological disorders receiving enteral nutrition and may adversely affect nutritional management and rehabilitation outcomes [15,16]. Constipation has also been recognized as a common yet often overlooked complication in patients with central neurological diseases, highlighting the importance of bowel assessment alongside nutritional management [17]. More recently, the BSFS has been incorporated into the assessment of enteral nutrition-associated gastrointestinal outcomes in patients with ischemic stroke [18]. The present analysis distinguished hard, normal, and soft or watery stool rather than combining abnormal stool forms. The association with hard versus normal stool persisted across the sequential models, whereas the association with soft or watery stool did not reach statistical significance after adjustment for fluid intake. This latter finding does not establish the absence of an association, given the uncertainty of the estimates. Evaluating stool-form categories separately therefore provided a more nuanced description of the observed relationships, which require confirmation in larger samples.

Recorded daily fluid intake was lower in the FOIS Level 1 group. However, participants at FOIS Level 1 had no oral intake, and recorded volumes excluded water contained in enteral formulas. The observed difference therefore partly reflected the feeding-group definitions and could not be interpreted as a difference in total water intake or hydration status. Because recorded fluid intake was closely related to oral intake status, its inclusion in Model 3 did not represent adjustment for an entirely independent covariate. The attenuation of the FOIS estimate after this adjustment does not establish that fluid intake explained or mediated the association with hard stool. Recorded fluid intake was not significantly associated with stool-form categories or BSFS score. Previous studies have reported associations between inadequate hydration, increased nutritional risk, and impaired bowel function in patients with neurological disorders [17]. Similarly, a recent scoping review identified reduced fluid intake, inadequate nutritional intake, and prolonged dependence on enteral nutrition as potential contributors to constipation in patients with dysphagia [19]. Although fibre-enriched enteral formulas may improve stool characteristics and reduce gastrointestinal complications in some patients, their effects on defecation frequency have been inconsistent across studies [20]. In the present study, defecation frequency differed between FOIS groups, but the ordinal regression model was not retained for inferential interpretation because of the violated proportional-odds assumption and sparse cells. Adjusted associations with defecation frequency were therefore not reported. These findings support considering bowel function alongside nutritional and fluid intake assessment, without attributing the observed differences to any single factor.

From a clinical perspective, bowel assessment may complement the evaluation of patients with neurogenic dysphagia alongside swallowing safety, aspiration risk, and nutritional adequacy. Oral health problems are also common among patients with stroke [21], further illustrating the need for multidimensional assessment. The observed differences in stool form and defecation frequency between FOIS groups suggest that bowel function warrants attention, although the present findings do not support treatment decisions based specifically on FOIS category or enteral feeding route. Simple tools, such as the BSFS and routine recording of defecation frequency, may help identify bowel-function concerns and prompt further nutritional and hydration assessment. This interpretation is consistent with studies emphasizing gastrointestinal tolerance and bowel function as relevant components of enteral nutrition monitoring [16,19].

Participants receiving PEG feeding were older than those receiving NG-tube feeding. No statistically significant between-group differences were detected in NRS-2002 score, enteral feeding duration, BSFS score, or daily fluid intake; however, these findings do not establish equivalence between the groups. The NG and PEG groups should be regarded as clinically distinct populations, and the revised analyses do not establish an independent association between feeding route and bowel outcomes. Previous studies have indicated that enteral access selection is guided by the expected duration of nutritional support and dysphagia severity [22]. Nutritional and clinical outcomes in patients with neurological disorders may also reflect dysphagia characteristics, nutritional status, and overall clinical condition [4,5,15]. Gastrointestinal complications may occur with either feeding route, emphasizing the importance of regular gastrointestinal monitoring during enteral nutritional support [16]. Larger studies with more comparable feeding-route groups and detailed assessment of clinical differences are needed to clarify potential associations with bowel function.

A strength of this study is the combined assessment of functional oral intake, stool form, defecation frequency, nutritional risk, daily fluid intake, and enteral feeding characteristics within the same cohort of adults with neurogenic dysphagia. Evaluating stool-form categories separately distinguished associations with hard stool from those with soft or watery stool. Sequential multinomial models allowed examination of changes in the FOIS estimate after adjustment for age and fluid intake, while Firth sensitivity analyses provided an alternative estimation approach for assessing the association with hard stool.

The association with hard versus normal stool persisted across the revised models. However, the association with soft or watery stool remained uncertain after adjustment for fluid intake; the nonsignificant result should not be interpreted as evidence of no association or as establishing that the findings were specific to hard stool. FOIS describes functional oral intake rather than physiological dysphagia severity. Participants classified as FOIS Level 1 had no oral intake, whereas those classified as Levels 2–3 continued to require tube feeding but had varying degrees of oral intake. These groups may therefore represent different clinical and nutritional profiles that cannot be made comparable through limited statistical adjustment.

Several limitations should be acknowledged. The cross-sectional design precludes causal or temporal inference, and reverse causality cannot be excluded. The single-center setting, small sample, and clinical heterogeneity limit generalizability. Restricting eligibility to participants with MMSE scores ≥ 24 also limits applicability to patients with greater cognitive impairment. Dementia subtype and stage were unavailable, limiting characterization of this subgroup. Sparse diagnostic categories contributed to unstable estimates in the initial complex models, prompting simpler adjusted analyses. Although Firth sensitivity analyses yielded similar findings, confidence intervals remained wide, and penalization does not resolve unmeasured confounding. No formal a priori sample-size calculation was performed. The sensitivity power analysis conducted during revision concerned the unadjusted comparison and did not establish adequate power for the final regression models. Multiple secondary comparisons were performed without multiplicity adjustment, increasing the possibility of chance findings, while limited precision means that potentially relevant associations may have remained undetected.

The ordinal regression model of defecation frequency violated the proportional-odds assumption and contained sparse cells; it was therefore not retained for inferential interpretation. Defecation-frequency findings were limited to descriptive summaries and unadjusted group comparisons. The sample size also limited reliable feeding-route subgroup analyses and assessment of interactions between FOIS group and feeding route. Complete screening and exclusion counts were unavailable, preventing reconstruction of a detailed participant flow.

Stool form, defecation frequency, and usual daily fluid intake were primarily participant-reported and cross-checked against available nursing records and feeding charts. Recall and reporting bias nevertheless remain possible. A fixed recall period was not specified for stool form or defecation frequency, potentially introducing variability in participants’ responses. Dysphagia duration was excluded from the reported analyses because its reference date could not be consistently verified from the available records. This did not alter the final regression models, which did not include this variable. Important potential confounders were unavailable or insufficiently standardized, including enteral formula composition, fibre content, feeding schedule and volume, chronic medication use, mobility, neurological disease severity, functional status, and pre-existing bowel habits. Consequently, pre-existing constipation could not be distinguished from bowel dysfunction developing during enteral feeding. Excluding recent changes in selected bowel-related treatments did not eliminate potential confounding from their continued use.

The amount, frequency, consistency, and nutritional contribution of oral intake among participants classified as FOIS Levels 2–3 were not quantified. Recorded fluid volumes included oral fluids and water administered through the feeding tube, including routine flushes, but excluded water contained in enteral formulas. This measure therefore did not represent total daily water intake or an objective assessment of hydration status, limiting interpretation of the contribution of fluid intake to bowel outcomes. Patient-specific indications for NG or PEG placement were also insufficiently documented. Differences between feeding-route groups may therefore reflect disease chronicity, expected duration of nutritional support, and overall clinical condition. These limitations prevent attribution of the observed bowel-function differences specifically to FOIS status or feeding route.

Future multicenter studies should recruit larger, more balanced samples and prospectively record bowel habits, nutritional intake, all sources of water intake, formula characteristics, medication use, mobility, and disease severity. Repeated assessments would help clarify temporal relationships, while analyses appropriate for sparse data and outcome distributions would improve estimation. Intervention studies are needed before concluding that changes in fluid provision, nutritional management, or swallowing rehabilitation improve bowel outcomes in this population.

## 5. Conclusions

In this cohort of enterally fed adults with neurogenic dysphagia, participants classified as FOIS Level 1 more frequently reported hard stool and less frequently reported normal stool than those classified as FOIS Levels 2–3. The association with hard versus normal stool persisted after adjustment for age and recorded daily fluid intake, with similar findings in Firth sensitivity analyses. Defecation frequency also differed between FOIS groups. Lower recorded fluid intake in the FOIS Level 1 group partly reflected the absence of oral intake and excluded water contained in enteral formulas. Attenuation of the FOIS estimate after fluid adjustment therefore does not establish that fluid intake explained the observed association.

These findings should be interpreted within the clinical context of the comparison groups. FOIS Level 1 and FOIS Levels 2–3 represent different functional feeding patterns rather than directly comparable clinical populations. Differences in oral intake, enteral formula composition, fibre exposure, medication use, mobility, functional status, and neurological disease severity may have contributed to the observed bowel outcomes. These factors were not measured in sufficient detail, and adjustment for age and fluid intake could not establish clinical comparability. Similarly, the clinical differences between NG and PEG groups prevent conclusions about an independent effect of feeding route.

The findings therefore remain exploratory and hypothesis-generating and do not establish that changing oral intake status or feeding route would improve bowel function. Nevertheless, they suggest that assessment of stool form and defecation frequency may complement routine swallowing and nutritional evaluation. Larger prospective multicenter studies incorporating detailed nutritional, fluid, medication, functional, and baseline bowel assessments are needed to clarify these relationships and their clinical implications.

## Figures and Tables

**Table 1:** Demographic, Clinical, Nutritional, and Bowel-Function Characteristics of Enterally Fed Adults with Neurogenic Oropharyngeal Dysphagia.

Characteristic	Category or Measure	*n* (%)	Mean ± SD	Min–Max
**Sex**	Female	34 (34.0)	–	–
	Male	66 (66.0)	–	–
**Clinical diagnosis**	Stroke	36 (36.0)	–	–
	Dementia	20 (20.0)	–	–
	Traumatic brain injury	22 (22.0)	–	–
	Parkinson’s disease	16 (16.0)	–	–
	Multiple sclerosis	6 (6.0)	–	–
**FOIS**	Level 1	55 (55.0)	–	–
	Level 2	24 (24.0)	–	–
	Level 3	21 (21.0)	–	–
**Enteral feeding route**	NG tube	41 (41.0)	–	–
	PEG	59 (59.0)	–	–
**Defecation frequency**	Daily	43 (43.0)	–	–
	Every 2–3 days	24 (24.0)	–	–
	Once or twice weekly	20 (20.0)	–	–
	Less than once weekly	13 (13.0)	–	–
**Stool-form classification**	Hard stool (BSFS Types 1–2)	38 (38.0)	–	–
	Normal stool (BSFS Types 3–4)	43 (43.0)	–	–
	Soft or watery stool (BSFS Types 5–7)	19 (19.0)	–	–
**Nutritional risk classification**	NRS-2002 < 3	19 (19.0)	–	–
	NRS-2002 ≥ 3	81 (81.0)	–	–
**Age**	Years	–	53.93 ± 17.30	19–86
**NRS-2002 score**	Score	–	3.85 ± 1.25	2–6
**Duration of enteral feeding**	Months	–	3.59 ± 1.17	2–6
**BSFS score**	Score	–	3.14 ± 1.64	1–7
**Daily fluid intake**	mL/day	–	1365.00 ± 375.68	800–2200

Data are presented as n (%), mean ± standard deviation (SD), or minimum–maximum. Percentages are calculated using the total sample (N = 100). FOIS Levels 2 and 3 were combined for comparative analyses (n = 45; 45.0%). An NRS-2002 score ≥ 3 indicates nutritional risk. Higher BSFS scores indicate progressively softer stool forms rather than better bowel function. Bold variable labels are used for readability only and do not indicate statistical significance. Abbreviations: FOIS, Functional Oral Intake Scale; NG, nasogastric; PEG, percutaneous endoscopic gastrostomy; NRS-2002, Nutritional Risk Screening 2002; BSFS, Bristol Stool Form Scale.

**Table 2:** Comparison of Age, Nutritional Risk, Enteral Feeding Duration, Stool Form, and Recorded Daily Fluid Intake between Functional Oral Intake Groups in Enterally Fed Adults with Neurogenic Oropharyngeal Dysphagia.

Variable	FOIS Level 1 (*n* = 55), Median (Q1–Q3)	FOIS Levels 2–3 (*n* = 45), Median (Q1–Q3)	Mann–Whitney U	*p*
Age (years)	61 (41–67)	59 (42–68)	1218.0	0.895
NRS-2002 score	4 (3–5)	4 (2–5)	1443.0	0.145
Duration of enteral feeding (months)	4 (3–5)	3 (2–4)	1711.5	**<0.001**
BSFS score	2 (1–4)	4 (3–4)	807.0	**0.002**
Daily fluid intake (mL/day)	1250 (1000–1475)	1400 (1200–1800)	912.0	**0.024**

Data are presented as median (first–third quartiles, Q1–Q3). Between-group comparisons were performed using the two-sided Mann–Whitney U test. Bold *p* values indicate statistical significance (*p* < 0.05). Lower BSFS scores indicate harder stool forms. Abbreviations: FOIS, Functional Oral Intake Scale; NRS-2002, Nutritional Risk Screening 2002; BSFS, Bristol Stool Form Scale.

**Table 3:** Comparison of Sex, Neurological Diagnosis, Enteral Feeding Route, Defecation Frequency, and Stool-Form Categories between Functional Oral Intake Groups in Enterally Fed Adults with Neurogenic Oropharyngeal Dysphagia.

Variable	Category	FOIS Level 1 (*n* = 55), n (%)	FOIS Levels 2–3 (n = 45), *n* (%)	Test	*p*
**Sex**	Female	20 (36.4)	14 (31.1)	Pearson χ^2^ = 0.304	0.581
	Male	35 (63.6)	31 (68.9)		
**Clinical diagnosis**	Stroke	19 (34.5)	17 (37.8)	Fisher–Freeman–Halton exact test	0.132
	Dementia	12 (21.8)	8 (17.8)		
	Traumatic brain injury	9 (16.4)	13 (28.9)		
	Parkinson’s disease	9 (16.4)	7 (15.6)		
	Multiple sclerosis	6 (10.9)	0 (0.0)		
**Enteral feeding route**	NG tube	17 (30.9)	24 (53.3)	Pearson χ^2^ = 5.145	**0.023**
	PEG	38 (69.1)	21 (46.7)		
**Defecation frequency**	Less than once weekly	6 (10.9)	7 (15.6)	Fisher–Freeman–Halton exact test	**0.003**
	Once or twice weekly	14 (25.5)	6 (13.3)		
	Every 2–3 days	19 (34.5)	5 (11.1)		
	Daily	16 (29.1)	27 (60.0)		
**Stool-form classification**	Hard stool (BSFS Types 1–2)	31 (56.4)	7 (15.6)	Fisher–Freeman–Halton exact test	**<0.001**
	Normal stool (BSFS Types 3–4)	13 (23.6)	30 (66.7)		
	Soft or watery stool (BSFS Types 5–7)	11 (20.0)	8 (17.8)		

Data are presented as n (%). Percentages were calculated within each FOIS group. Between-group comparisons were performed using Pearson’s chi-square test or the Fisher–Freeman–Halton exact test, as appropriate. Each *p* value refers to the overall comparison for the corresponding variable. Bold variable labels are used for readability only and do not indicate statistical significance. Bold *p* values indicate statistical significance (*p* < 0.05). Abbreviations: FOIS, Functional Oral Intake Scale; NG, nasogastric; PEG, percutaneous endoscopic gastrostomy; BSFS, Bristol Stool Form Scale.

**Table 4:** Recorded Daily Fluid Intake by Functional Oral Intake Group and Stool-Form Category and Its Correlation with Bristol Stool Form Scale Score in Enterally Fed Adults with Neurogenic Oropharyngeal Dysphagia.

Analysis	Group or Association	*n*	Daily Fluid Intake (mL/Day), Mean ± SD	Spearman’s ρ	*p*
**FOIS group**	Level 1	55	1280.0 ± 326.1	–	**0.024**
	Levels 2–3	45	1468.9 ± 408.6	–	
**Stool-form category**	Hard stool (BSFS Types 1–2)	38	1253.9 ± 323.9	–	0.098
	Normal stool (BSFS Types 3–4)	43	1450.0 ± 432.0	–	
	Soft/watery stool (BSFS Types 5–7)	19	1394.7 ± 284.3	–	
**Correlation**	Daily fluid intake and BSFS score	100	–	0.131	0.194

Daily fluid intake is presented as mean ± standard deviation (SD). Recorded daily fluid intake excluded water contained in enteral formulas. Between-group comparisons were performed using the Mann–Whitney U test for FOIS groups and the Kruskal–Wallis test for stool-form categories. Each group-comparison *p* value refers to the overall comparison for the corresponding grouping variable. The association between daily fluid intake and BSFS score was assessed using Spearman’s rank correlation coefficient (ρ). Higher BSFS scores indicate progressively softer stool forms rather than better bowel function. These analyses were exploratory. Bold analysis labels are used for readability only and do not indicate statistical significance. Bold *p* values indicate nominal statistical significance (*p* < 0.05). Abbreviations: FOIS, Functional Oral Intake Scale; BSFS, Bristol Stool Form Scale.

**Table 5:** Clinical Characteristics by Enteral Feeding Route in Adults with Neurogenic Oropharyngeal Dysphagia.

Variable	NG Tube (*n* = 41), Median (Q1–Q3)	PEG (*n* = 59), Median (Q1–Q3)	*p*
Age (years)	53 (32–63)	61 (45.5–69.5)	**0.034**
NRS-2002 score	4 (3–4)	4 (3–5)	0.903
Duration of enteral feeding (months)	4 (2–5)	3 (3–4)	0.896
BSFS score	3 (2–4)	3 (1–5)	0.476
Daily fluid intake (mL/day)	1200 (1000–1500)	1400 (1100–1725)	0.269

Data are presented as median (first–third quartiles, Q1–Q3). Recorded daily fluid intake excluded water contained in enteral formulas. Between-group comparisons were performed using the two-sided Mann–Whitney U test. Bold *p* values indicate statistical significance (*p* < 0.05). Abbreviations: NG, nasogastric; PEG, percutaneous endoscopic gastrostomy; NRS-2002, Nutritional Risk Screening 2002; BSFS, Bristol Stool Form Scale.

**Table 6:** Multicollinearity Diagnostics for Predictors in the Multinomial Logistic Regression Model of Stool Form.

Variable	Tolerance	VIF
FOIS Level 1 vs. Levels 2–3	0.934	1.070
Age	0.954	1.048
Daily fluid intake, per 100 mL/day	0.894	1.119

Diagnostics were calculated from the participant-level dataset using the final model predictor set (N = 100). Each predictor was regressed on the remaining predictors using an auxiliary linear regression with an intercept. VIF < 5 and tolerance > 0.20 were used as reference thresholds. Abbreviations: FOIS, Functional Oral Intake Scale; VIF, variance inflation factor.

**Table 7:** Multinomial Logistic Regression Models of Functional Oral Intake and Stool-Form Categories in Adults with Neurogenic Dysphagia.

Model/Variable	Hard vs. Normal Stool, OR (95% CI)	*p*	Soft/Watery vs. Normal Stool, OR (95% CI)	*p*
**Model 1: FOIS group**				
FOIS Level 1 vs. Levels 2–3	10.22 (3.59–29.12)	**<0.001**	3.17 (1.04–9.72)	**0.043**
**Model 2: FOIS group + age**				
FOIS Level 1 vs. Levels 2–3	10.33 (3.61–29.55)	**<0.001**	3.31 (1.06–10.34)	**0.040**
Age, per year	1.008 (0.980–1.038)	0.568	1.028 (0.993–1.065)	0.119
**Model 3: FOIS group + age + fluid intake**				
FOIS Level 1 vs. Levels 2–3	8.93 (3.07–25.97)	**<0.001**	3.13 (0.98–9.98)	0.053
Age, per year	1.015 (0.985–1.046)	0.341	1.030 (0.994–1.067)	0.106
Daily fluid intake, per 100 mL/day	0.887 (0.763–1.032)	0.121	0.964 (0.822–1.131)	0.655

Normal stool (BSFS Types 3–4) was the reference outcome category; hard stool comprised Types 1–2 and soft/watery stool comprised Types 5–7. FOIS Levels 2–3 were the reference FOIS group. All models included 100 participants. ORs represent the odds of the specified stool-form category relative to normal stool. Recorded daily fluid intake excluded water contained in enteral formulas. Bold model headings are used for readability only and do not indicate statistical significance. Bold *p* values indicate nominal statistical significance (*p* < 0.05). Abbreviations: FOIS, Functional Oral Intake Scale; BSFS, Bristol Stool Form Scale; OR, odds ratio; CI, confidence interval.

**Table 8:** Firth Logistic Regression Sensitivity Analyses of Functional Oral Intake and Hard Stool in Adults with Neurogenic Dysphagia.

Outcome	Model	Variable	OR (95% CI)	*p*	N
Hard vs. normal stool	Unadjusted	FOIS Level 1 vs. Levels 2–3	9.49 (3.56–27.95)	**<0.001**	81
Hard vs. normal stool	Age-adjusted	FOIS Level 1 vs. Levels 2–3	9.38 (3.51–27.93)	**<0.001**	81
		Age, per 10 years	0.964 (0.709–1.298)	0.808	
Hard vs. all other stool forms	Age-adjusted	FOIS Level 1 vs. Levels 2–3	6.46 (2.64–17.51)	**<0.001**	100
		Age, per 10 years	0.986 (0.764–1.275)	0.916	

Firth-penalized logistic regression was used for sensitivity analyses. Confidence intervals were calculated using the profile-likelihood method. For the hard versus normal stool analyses, participants with soft/watery stool were excluded (N = 81). The hard versus all other stool forms analysis included all participants (N = 100). FOIS Levels 2–3 were the reference group. Clinical diagnosis was not included because of sparse diagnostic categories. Bold *p* values indicate nominal statistical significance (*p* < 0.05). Abbreviations: FOIS, Functional Oral Intake Scale; OR, odds ratio; CI, confidence interval.

## Data Availability

The data supporting the findings of this study are available from the corresponding author upon reasonable request.

## Supplementary Materials

The following supporting information can be downloaded at: https://www.mdpi.com/article/10.3390/jcm15218529/s1, Supplementary File S1: STROBE checklist.
