# Supplementary material for: Functional Oral Intake and Bowel Function in Enterally Fed Adults with Neurogenic Dysphagia: A Cross-Sectional Study
Source: J Clin Med. 2026 Nov 2;15(21):8529. doi: 10.3390/jcm15218529 (PMC13763758; doi:10.3390/jcm15218529)
Supplement: Supplementary file 1 [file jcm-15-08529-s001.zip › jcm-4639254-supplementary.pdf]

Functional Oral Intake, Nutritional Risk and Bowel Function in Enterally Fed Adults with Neurogenic Dysphagia: A Cross-Sectional Study

STROBE Statement—checklist of items that should be included in reports of observational studies

Page references count the supplied manuscript from its first title/author page (p. 1); Abstract is p. 4. Section and table locators accompany the page references. Not reported and partially reported items are distinguished from items that do not apply.

	No.	Recommendation	Reported in manuscript / status
**Title and abstract**	1	(*a*) Indicate the study's design with a commonly used term in the title or the abstract	Title (pp. 1 and 4) identifies the study as "A Cross-Sectional Study"; Abstract (p. 4) describes a prospective cross-sectional design.
		(*b*) Provide in the abstract an informative and balanced summary of what was done and what was found	Abstract (p. 4): summarizes the objective, design, 100 participants, FOIS, NRS-2002, BSFS, analyses, principal findings and an adjusted OR with 95% CI. It identifies the ordinal-model assumption violation and gives cautious, hypothesis-generating conclusions.
**Introduction**			
Background/rationale	2	Explain the scientific background and rationale for the investigation being reported	1. Introduction (p. 5): explains neurogenic dysphagia, enteral nutrition and gastrointestinal complications, and the limited evidence relating functional oral intake and nutritional risk to stool consistency and defecation patterns.
Objectives	3	State specific objectives, including any prespecified hypotheses	1. Introduction, final paragraph (pp. 5–6): states the association and feeding-route comparison objectives, and hypothesizes that lower oral intake and greater nutritional risk relate to hard stool and lower defecation frequency. Comparisons are explicitly descriptive, not causal.
**Methods**			
Study design	4	Present key elements of study design early in the paper	2.1 Study Design and Setting (p. 6): identifies the prospective cross-sectional observational design, clinical setting, recruitment and data sources early in Methods. 2.4 (pp. 8–9) describes a single assessment session whenever possible.
Setting	5	Describe the setting, locations, and relevant dates, including periods of recruitment, exposure, follow-up, and data collection	2.1 (p. 6): Neurology and Physical Medicine and Rehabilitation clinics, Istanbul Atlas University Hospital; data collection during May–July 2026 after ethics approval on 11 May. 2.2 (p. 7): recruitment during the study period. Longitudinal follow-up: not applicable.
Participants	6	(*a*) *Cohort study*—Give the eligibility criteria, and the sources and methods of selection of participants. Describe methods of follow-up<br>*Case-control study*—Give the eligibility criteria, and the sources and methods of case ascertainment and control selection. Give the rationale for the choice of cases and controls<br>*Cross-sectional study*—Give the eligibility criteria, and the sources and methods of selection of participants	2.2 Study Population (pp. 6–8): specifies source clinics, neurological diagnoses, prior clinical and VFSS/FEES confirmation, age ≥18, NG/PEG feeding for ≥1 month, clinical stability, MMSE ≥24 and exclusions. Recruitment is described as both consecutive and convenience sampling; this ambiguity is not resolved in the manuscript.
		(*b*) *Cohort study*—For matched studies, give matching criteria and number of exposed and unexposed<br>*Case-control study*—For matched studies, give matching criteria and the number of controls per case	Not applicable: this cross-sectional study did not report a matched cohort or case-control design.

	No.	Recommendation	Reported in manuscript / status
Variables	7	Clearly define all outcomes, exposures, predictors, potential confounders, and effect modifiers. Give diagnostic criteria, if applicable	2.2–2.5 (pp. 6–13): defines dysphagia eligibility, FOIS groups, NRS-2002, BSFS, defecation-frequency categories, fluid intake, clinical diagnoses and feeding characteristics; identifies model covariates. FOIS reflects feeding status, not physiological dysphagia severity. Important unmeasured factors are acknowledged in Discussion (pp. 20–22).
Data sources/ measurement	8*	For each variable of interest, give sources of data and details of methods of assessment (measurement). Describe comparability of assessment methods if there is more than one group	2.3–2.4.6 (pp. 8–11): standardized assessments by the same researcher, participant interviews and medical records; nursing/feeding records used for verification when available. Describes scale scoring and common procedures across groups. Oral-intake quantity was not measured; a fixed recall interval for usual bowel habits and operational definition of dysphagia duration are not specified.
Bias	9	Describe any efforts to address potential sources of bias	2.2–2.5 (pp. 7–13): eligibility restrictions, exclusion of recent bowel-medication changes, standardized assessments, record verification and adjustment for measured covariates. Discussion (pp. 19–22) addresses non-comparable groups, unmeasured confounding, recall/reporting bias and reverse causality.
Study size	10	Explain how the study size was arrived at	2.2 Study Population (pp. 7–8): G*Power 3.1, $f^2 = 0.15$, $\alpha = 0.05$ and 80% power yielded n = 84; target increased to ≥100 for incomplete data. The calculation was not for the fully adjusted multinomial model. The exact test and number of predictors used in the calculation are not specified.
Quantitative variables	11	Explain how quantitative variables were handled in the analyses. If applicable, describe which groupings were chosen and why	2.4.1–2.5 (pp. 9–13): means, SDs and ranges; FOIS 1 versus 2–3; BSFS 1–2, 3–4 and 5–7; four ordered defecation categories; fluid intake per 100 mL/day. Explains the feeding-status grouping and why stool form requires nominal categories rather than a favourable-to-unfavourable ordinal outcome.
Statistical methods	12	(*a*) Describe all statistical methods, including those used to control for confounding	2.5 Statistical Analysis (pp. 11–13): t-tests, chi-square tests, Spearman correlations, sequential multinomial models and ordinal regression. Model 2 adjusts for age/diagnosis; Model 3 adds dysphagia duration, NRS-2002, feeding duration, fluid intake and route. Covariates were chosen a priori for clinical relevance. Reference categories, diagnostics, 95% CIs and two-sided $p < 0.05$ are specified.
		(*b*) Describe any methods used to examine subgroups and interactions	2.5 (p. 11): t-tests compare FOIS and feeding-route groups; chi-square tests compare categorical variables by FOIS. Discussion (p. 21): route-specific subgroup analyses and FOIS × route interaction could not be reliably evaluated because of small, sparse groups; these analyses were not performed.

	No.	Recommendation	Reported in manuscript / status
		(*c*) Explain how missing data were addressed	2.2 (p. 7) excludes incomplete clinical data. 2.5 (p. 13) reports completeness checks and no missing dependent or independent regression variables; all regression models use n = 100. Table 7 notes (p. 35) confirm this. Numbers excluded for incomplete data are not reported.
		(*d*) *Cohort study*—If applicable, explain how loss to follow-up was addressed<br>*Case-control study*—If applicable, explain how matching of cases and controls was addressed<br>*Cross-sectional study*—If applicable, describe analytical methods taking account of sampling strategy	2.2 (p. 7) describes consecutive recruitment using convenience sampling. 2.5 (pp. 11–13) reports no sampling weights, clustering or other design-specific adjustment. The recruitment terminology is ambiguous. Follow-up and matched case-control adjustments: not applicable.
		(*e*) Describe any sensitivity analyses	Not reported as sensitivity analyses. 2.5 (p. 12), 3.7 (p. 16) and Table 7 (pp. 33–36) describe prespecified sequential adjustment models to assess changes in the FOIS estimate; no separate sensitivity analysis is identified.
**Results**			
Participants	13*	(a) Report numbers of individuals at each stage of study—eg numbers potentially eligible, examined for eligibility, confirmed eligible, included in the study, completing follow-up, and analysed	Partially reported: 3.1 (pp. 13–14) reports 100 included participants; 2.5 (p. 13) confirms all 100 in regression analyses. Numbers screened, potentially eligible, excluded or declining participation are not reported. Follow-up completion: not applicable.
		(b) Give reasons for non-participation at each stage	Not reported: numbers and reasons for non-participation at each recruitment stage are not given. The exclusion criteria in 2.2 (p. 7) do not provide actual exclusion counts or participant-specific reasons.
		(c) Consider use of a flow diagram	Not included: no participant flow diagram is provided. The manuscript reports the final sample but not the preceding recruitment flow.
Descriptive data	14*	(a) Give characteristics of study participants (eg demographic, clinical, social) and information on exposures and potential confounders	3.1 (pp. 13–14) and Table 1 (pp. 27–28): demographics, diagnoses, FOIS, route, nutritional risk, feeding/dysphagia duration, fluid intake and bowel outcomes. Tables 2–3 (pp. 28–30) provide FOIS-group data; Table 5 (pp. 31–32) compares clinical characteristics by feeding route. Unmeasured confounders are acknowledged on pp. 21–22.
		(b) Indicate number of participants with missing data for each variable of interest	2.5 (p. 13) states no missing values in any dependent or independent regression variable; Table 7 notes (p. 35) confirm n = 100 for each model. Table 1 (p. 28) reports N = 100. A separate variable-by-variable missing-data table is not provided.
		(c) *Cohort study*—Summarise follow-up time (eg, average and total amount)	Not applicable: this cross-sectional study had no longitudinal follow-up period. Dysphagia and enteral feeding durations are clinical characteristics, not follow-up time.
Outcome data	15*	*Cohort study*—Report numbers of outcome events or summary measures over time	Not applicable: this was not a cohort study and did not report outcome events over follow-up.

	No.	Recommendation	Reported in manuscript / status
		Case-control study—Report numbers in each exposure category, or summary measures of exposure	Not applicable: this was not a case-control study; participants were not selected as cases and controls.
		Cross-sectional study—Report numbers of outcome events or summary measures	3.1 (pp. 13–14), Table 1 (p. 28) and Table 3 (p. 30): hard/normal/soft-watery stool in 38/43/19 participants; defecation daily/every 2–3 days/once or twice weekly/less than weekly in 43/24/20/13. FOIS-group outcome distributions and BSFS summary measures are reported.
Main results	16	(*a*) Give unadjusted estimates and, if applicable, confounder-adjusted estimates and their precision (eg, 95% confidence interval). Make clear which confounders were adjusted for and why they were included	3.7–3.8 (pp. 16–17), Tables 7–8 (pp. 33–37): unadjusted and adjusted ORs with 95% CIs; unstable or non-estimable results are marked. FOIS/hard-stool OR: 8.158 unadjusted, 38.403 fully adjusted. Covariates and clinical rationale: 2.5 (p. 12). Tables 2–5 (pp. 28–32) give unadjusted comparisons/correlations without 95% CIs.
		(*b*) Report category boundaries when continuous variables were categorized	2.4.1–2.5 (pp. 9–12) and Tables 1, 3 (pp. 28, 30): boundaries for grouped scale scores are FOIS 1 versus 2–3 and BSFS 1–2/3–4/5–7; defecation categories are explicit. Continuous covariates were not categorized in regression. Fluid intake was rescaled per 100 mL/day, not categorized.
		(*c*) If relevant, consider translating estimates of relative risk into absolute risk for a meaningful time period	Not applicable: the analyses estimate cross-sectional odds ratios, not relative risks over a follow-up period. No time-dependent absolute risk was estimated.
Other analyses	17	Report other analyses done—eg analyses of subgroups and interactions, and sensitivity analyses	3.2–3.6 (pp. 14–15), Tables 2–6 (pp. 28–33): FOIS/feeding-route comparisons, Spearman correlations and multicollinearity diagnostics. Sequential adjustment: 3.7 (p. 16), Table 7 (pp. 33–36). Route-specific subgroup/interaction analyses were not performed (p. 21); separate sensitivity analyses are not reported.
**Discussion**			
Key results	18	Summarise key results with reference to study objectives	4. Discussion, opening paragraph (p. 17), and 5. Conclusion (pp. 22–23): summarize the stool-form and defecation-frequency associations in relation to study objectives, with warnings about imprecision and exploratory models.
Limitations	19	Discuss limitations of the study, taking into account sources of potential bias or imprecision. Discuss both direction and magnitude of any potential bias	4. Discussion (pp. 19–22): non-comparable groups, cross-sectional temporality/reverse causality, single-centre selection, small heterogeneous sample, sparse cells, wide CIs, separation, violated proportional odds, self-report and unmeasured nutritional/clinical factors. Possible bias mechanisms are discussed, but their direction and magnitude are not systematically estimated.
Interpretation	20	Give a cautious overall interpretation of results considering objectives, limitations, multiplicity of analyses, results from similar studies, and other relevant evidence	4. Discussion (pp. 17–22) and 5. Conclusion (pp. 22–23): relates findings to prior evidence, acknowledges model and measurement limitations, avoids causal/independent-effect claims and labels results exploratory. The implications of multiple testing are not explicitly discussed; no multiplicity adjustment is reported in 2.5 (pp. 11–13).

	No.	Recommendation	Reported in manuscript / status
Generalisability	21	Discuss the generalisability (external validity) of the study results	4. Discussion, limitations and future directions (pp. 21–22): single-centre recruitment and a small, clinically heterogeneous sample limit generalisability; larger, balanced multicentre studies are proposed. 5. Conclusion (pp. 22–23) emphasizes the clinical differences between the comparison groups.
**Other information**			
Funding	22	Give the source of funding and the role of the funders for the present study and, if applicable, for the original study on which the present article is based	Funding (p. 23): "This research received no external funding." An external funder role is therefore not applicable; no separately funded original study is described.

*Give information separately for cases and controls in case-control studies and, if applicable, for exposed and unexposed groups in cohort and cross-sectional studies.

Note: An Explanation and Elaboration article discusses each checklist item and gives methodological background and published examples of transparent reporting. The STROBE checklist is best used in conjunction with this article (freely available on the Web sites of PLoS Medicine at http://www.plosmedicine.org/, Annals of Internal Medicine at http://www.annals.org/, and Epidemiology at http://www.epidem.com/). Information on the STROBE Initiative is available at www.strobe-statement.org.
